# Trastuzumab Deruxtecan-Associated Interstitial Lung Disease: Real-World Insights from a Tertiary Care Center

**DOI:** 10.3390/curroncol32100575

**Published:** 2025-10-16

**Authors:** Ahmed S. Alanazi, Ahmed A. Alanazi, Abdalrhman Alanizi, Ranad Babalghaith, Reema Alotaibi, Mohammed Alnuhait, Hatoon Bakhribah

**Affiliations:** 1 Pharmacy Service Administration, King Fahad Medical City, Riyadh 11525, Saudi Arabia; asalalanazi@kfmc.med.sa; 2 Department of Pharmacy Practice, College of Pharmacy, Imam Abdulrahman Bin Faisal University, P.O. Box 1982, Dammam 31441, Saudi Arabia; aasalanazi@iau.edu.sa; 3 Pharmaceutical Care Service, King Abdullah Bin Abdulaziz University Hospital, Riyadh 11564, Saudi Arabia; ahalanizi@kaauh.edu.sa; 4 College of Pharmacy, Princess Nourah Bint Abdulrahman University, Riyadh 11671, Saudi Arabia; 5 Department of Clinical Pharmacy, College of Pharmacy, Shaqra University, Al-Dawadmi Campus, Al-Dawadmi 11961, Saudi Arabia; 6 Comprehensive Cancer Center, King Fahad Medical City, Riyadh 11525, Saudi Arabia; hbakharibah@kfmc.med.sa

**Keywords:** trastuzumab deruxtecan, interstitial lung disease, real-world data, HER2-positive cancer, pulmonary toxicity

## Abstract

**Simple Summary:**

Trastuzumab deruxtecan is a new cancer treatment that targets tumors carrying a protein called HER2. It has shown great promise for people with advanced cancers, especially breast cancer, but it can sometimes cause a serious lung condition called interstitial lung disease. This condition involves inflammation or scarring of the lungs and can lead to cough, shortness of breath, or, in rare cases, severe breathing problems. Because most large international trials did not include patients from the Middle East, it has been unclear whether the same risks apply to our population. In this study, we reviewed the medical records of 65 patients who received trastuzumab deruxtecan at a major hospital in Saudi Arabia. About one in four patients developed lung complications, most often within the first six months of treatment. The most common early warning signs were cough and shortness of breath. Patients who developed more serious lung complications had lower oxygen levels at the time of diagnosis. Most patients improved with prompt treatment, and there were no deaths in our group. These results highlight the importance of careful monitoring so that any lung side effects can be detected early and managed safely while patients continue to benefit from this important therapy.

**Abstract:**

**Background:** Trastuzumab deruxtecan (T-DXd), a HER2-directed antibody-drug conjugate, has significantly advanced the management of HER2-expressing malignancies. However, interstitial lung disease (ILD) remains a clinically significant adverse effect. Despite increasing clinical use of T-DXd, real-world data on ILD incidence, characteristics, and outcomes—particularly in Middle Eastern populations remain limited. **Methods:** This retrospective study analyzed medical records of patients who received trastuzumab deruxtecan (T-DXd) at a tertiary care hospital. Data collected included demographics, tumor characteristics, prior treatments, and interstitial lung disease (ILD)-related outcomes. ILD events were identified and graded according to the Common Terminology Criteria for Adverse Events (CTCAE) version 5.0. Descriptive statistics were used to summarize baseline characteristics and ILD features. Univariate logistic regression was performed to assess potential risk factors associated with ILD development. Kaplan–Meier survival analysis was used to evaluate time-to-event outcomes, including time to ILD onset and resolution. **Results:** Among 65 patients with advanced stage IV cancer (90.8% with breast cancer), 16 (24.6%) developed ILD following T-DXd therapy. The median time to ILD onset was 125.5 days. The most common presenting symptoms were dyspnea and cough (50%). A history of ground-glass opacities was associated with increased odds of ILD (OR 2.7; *p* = 0.236), though not statistically significant. Patients with Grade ≥ 3 ILD had significantly lower oxygen saturation levels compared to those with milder grades (88.3% vs. 97.7%, *p* = 0.049). Median time to clinical resolution was 297 days (95% CI: 77.5–516). No significant associations were observed with smoking history, pulmonary metastases, or prior thoracic radiation. **Conclusions:** In this real-world cohort, ILD occurred in nearly one-quarter of patients receiving T-DXd, predominantly within the first six months of treatment. The findings highlight the importance of early respiratory symptom monitoring and pulse oximetry—particularly in patients with pre-existing pulmonary abnormalities. These results underscore the need for vigilant ILD surveillance strategies and further prospective studies to validate predictive risk factors and optimize management protocols.

## 1. Introduction

Trastuzumab deruxtecan (T-DXd) is a HER2-directed antibody-drug conjugate (ADC) that has demonstrated significant efficacy in treating HER2-positive and HER2-low cancers, including metastatic breast cancer, gastric cancer, and non-small-cell lung cancer (NSCLC) [1,2]. It is important to distinguish between HER2-positive or HER2-low tumors, defined by protein expression, and HER2-mutant tumors, which are genomically characterized (most often in NSCLC). ILD incidence appears higher in HER2-mutant NSCLC compared with HER2-expressing breast cancers. Our study cohort comprised HER2-expressing cancers (HER2-positive and HER2-low) but did not include HER2-mutant NSCLC [1,2]. T-DXd consists of a humanized anti-HER2 monoclonal antibody linked to a topoisomerase I inhibitor payload via a cleavable linker. This design allows for potent cytotoxicity and a bystander killing effect, enhancing its activity even in tumors with heterogeneous or low HER2 expression [3]. However, with increased clinical use, one of the most serious and potentially fatal toxicities associated with T-DXd has emerged interstitial lung disease (ILD) [4]. ILD comprises a spectrum of pulmonary disorders characterized by inflammation and/or fibrosis of the lung interstitium. Clinically, it may present with symptoms such as dyspnea, cough, fatigue, or hypoxia, and radiologically as ground-glass opacities, reticulations, or organizing pneumonia patterns. Severe or untreated ILD can lead to respiratory failure and death [5]. The mechanism underlying T-DXd-induced ILD remains incompletely understood but is believed to involve several factors. These include direct alveolar epithelial cell damage from the cytotoxic payload, immune-mediated responses triggered by the ADC, and possibly accumulation in lung tissue via off-target uptake or Fc-mediated binding [6]. Clinical trials have consistently highlighted ILD as a major adverse event associated with T-DXd. In the pivotal DESTINY-Breast01 trial, ILD was reported in 13.6% of patients, including four treatment-related deaths [1] DESTINY-Breast03 a Phase III trial comparing T-DXd with trastuzumab emtansine (T-DM1), reported ILD in 10.5% of patients receiving T-DXd, compared to 1.9% in the T-DM1 arm [2]. In DESTINY-Lung01, which studied patients with HER2-mutant NSCLC, the incidence of ILD was notably higher at 26%, with several fatal outcomes [7]. A pooled safety analysis of nine clinical trials, encompassing over 1100 patients treated with T-DXd, reported an overall ILD incidence of 12.1% (95% CI: 9.9–14.6%), with Grade 5 (fatal) events in approximately 2.2% of cases. Notably, ILD rates have been shown to be dose-dependent, with the highest incidence observed at the 6.4 mg/kg dose tested in early phase trials, compared with lower approved dosing regimens. The median time to ILD onset was 5.4 months [4]. Risk factors identified in clinical trials and post hoc analyses include older age, reduced baseline oxygen saturation, prior or concurrent thoracic radiation, pre-existing pulmonary conditions, higher cumulative exposure to T-DXd, and Asian ethnicity [4,8]. These associations have been inconsistent across studies; therefore, in our analysis they were treated as exploratory rather than prespecified predictors. Real-world data have started to confirm and expand on findings from clinical trials. A recent United States-based retrospective multicenter study involving 314 patients reported ILD in 9.2% of patients treated with T-DXd in routine clinical practice, with 1.6% experiencing fatal outcomes [9].

The clinical presentation of T-DXd-induced ILD is variable. Mild cases may be asymptomatic and identified only through imaging, while more severe cases present with progressive respiratory symptoms and can deteriorate rapidly if untreated [10]. Management depends on ILD grade; treatment interruption and close monitoring are generally advised for Grade 1, while higher grades warrant permanent discontinuation of T-DXd and prompt initiation of corticosteroids. However, there remains no standardized steroid regimen for treatment, and institutional practices vary widely [8]. Real-world evidence is critical in capturing data from broader patient populations and can better inform clinical decision-making, risk stratification, and management strategies [11]. To date, no published studies have evaluated the incidence or management of T-DXd-induced ILD in Saudi Arabia or the Gulf region. This is an important gap, particularly given that patients from this geographic area were not represented in pivotal T-DXd trials. Ethnic and genetic differences may influence susceptibility to ILD, drug metabolism, or immune-related adverse events [8]. Given these considerations, the generation of locally derived real-world data is crucial for characterizing the presentation and management of T-DXd-induced ILD in Saudi patients, as well as for guiding the development of context-specific clinical guidelines and strengthening pharmacovigilance practices.

## 2. Methods

### 2.1. Study Design and Setting

This retrospective cohort study was conducted at King Fahad Medical City (KFMC), a tertiary care referral center located in Riyadh, Saudi Arabia. The study utilized real-world data extracted from the hospital’s electronic health records (EHR) to evaluate clinical outcomes related to trastuzumab deruxtecan (T-DXd) induced interstitial lung disease (ILD) among patients with advanced solid tumors.

### 2.2. Study Population

The study included all adult patients (≥18 years) who received at least one dose of T-DXd between January 2021 and January 2024 at KFMC. Patients were identified through the institutional pharmacy records and verified using oncology clinic documentation. Inclusion criteria were: (1) a confirmed diagnosis of HER2-expressing (HER2-positive or HER2-low) advanced or metastatic cancer. Patients with HER2-mutant NSCLC were not included. Patients were excluded if they had incomplete clinical documentation or if ILD diagnosis could not be reliably verified through imaging and clinical assessment.

### 2.3. Data Collection

Data were extracted from the electronic health record (EHR) system by trained data abstractors using a standardized and pre-defined data collection tool. The variables collected included patient demographics (age, sex, body mass index [BMI], and smoking history); cancer-related information such as the primary tumor type, HER2 status, stage at diagnosis, prior treatments including chemotherapy, radiotherapy, and immunotherapy, the presence of lung metastases, and relevant comorbidities. Treatment-related data for trastuzumab deruxtecan (T-DXd) included the line of therapy, the number of treatment cycles received, and any dose modifications. Cumulative exposure was also recorded, as T-DXd is typically administered until progression or unacceptable toxicity. ILD-related variables encompassed the timing of ILD onset relative to the first dose of T-DXd, presenting respiratory symptoms (e.g., dyspnea and cough), radiological findings including ground-glass opacities, and the severity of ILD as graded according to the Common Terminology Criteria for Adverse Events (CTCAE) version 5.0. Additional clinical data included oxygen saturation levels at ILD diagnosis, need for hospitalization, corticosteroid use, and outcomes such as clinical resolution or death. Duration of therapy and clinical response were also collected where available from medical records.

### 2.4. Outcome Measures

The primary outcome was the incidence of ILD during T-DXd therapy. Secondary outcomes included time to ILD onset, symptom severity, SpO_2_ at presentation, duration to clinical resolution, and the association between baseline characteristics and ILD development. Exploratory efficacy outcomes included overall survival (OS) and time-to-treatment-discontinuation.

### 2.5. Statistical Analysis

Descriptive statistics were used to summarize patient demographics and clinical characteristics. Continuous variables were presented as mean ± standard deviation (SD) or median with interquartile range (IQR), depending on distribution. Categorical variables were summarized using frequencies and percentages. Univariate logistic regression was performed to assess associations between patient- and disease-related factors and the development of ILD. The time to ILD onset and time to resolution were analyzed using Kaplan–Meier survival estimates. A *p*-value of <0.05 was considered statistically significant. All statistical analyses were conducted using SPSS software, version 26.0 (IBM Corp., Armonk, NY, USA).

### 2.6. Ethical Considerations

The study was conducted in accordance with the Declaration of Helsinki and was approved by the Research Ethics Committee of King Fahad Medical City (25-108). As this was a retrospective study utilizing electronic medical records, the requirement for informed consent was waived by the Ethics Committee.

## 3. Results

A total of 65 patients were included in this retrospective study. The mean age was 49 ± 11.82 years (range: 24–74), with the vast majority being female (*n* = 62, 95.4%) and only 3 male patients (4.6%). The cohort had an average body mass index (BMI) of 25.78 ± 5.75 kg/m^2^. The mean height was 156.76 ± 15.16 cm, and the average body weight was 63.37 ± 15.16 kg. All patients had advanced-stage (Stage IV) malignancies. The predominant cancer type was breast cancer (*n* = 59, 90.8%), followed by endometrial cancer (*n* = 2, 3.1%). HER2-positive status was observed in 38 patients (58.5%), and 56.3% had HER2 3+ by immunohistochemistry (IHC). In terms of hormone receptor status, 52.5% were progesterone receptor (PR) positive, and 67.8% were estrogen receptor (ER) positive. The average Ki-67 proliferation index was 41.68 ± 24.40%. Further details related to demographic and tumor characteristics are summarized in Table 1A. With regard to baseline medical and clinical risk factors, only 1.5% of patients reported a history of smoking, while over 98% were non-smokers. Ground-glass opacities were noted in baseline imaging in 60% of the patients. Additionally, 67% of the cohort had no documented history of allergies or drug contraindications. The most common sites of metastasis were the lung, liver, and brain (38.5%). Baseline oxygen saturation levels were generally well-preserved, with a mean SpO_2_ of 98.09 ± 1.77%, suggesting stable pulmonary function prior to T-DXd initiation. These findings are detailed in Table 1B.

### 3.1. Incidence of Interstitial Lung Disease (ILD)

**Figure 1:**
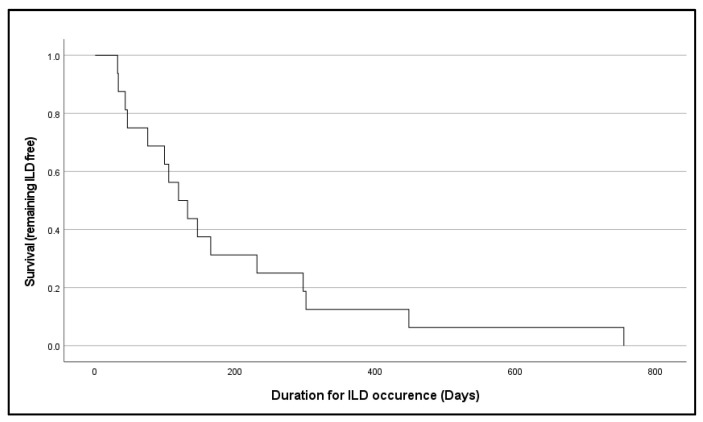
A Kaplan–Meier curve of ILD-free survival following T-DXd therapy initiation. ILD-free survival was defined as the time from T-DXd initiation to ILD diagnosis or last follow-up.

Out of 65 patients who received T-DXd, 16 (24.6%) developed ILD or pneumonitis, while the remaining 49 patients (75.4%) did not. We assessed whether ILD incidence was influenced by known risk factors, including smoking status, the presence of lung metastases, and prior thoracic radiotherapy. None of these factors showed a statistically significant association with ILD occurrence (*p* > 0.05) (Table 2). Kaplan–Meier analysis demonstrated a gradual decline in ILD-free survival following T-DXd initiation. Ten patients remained ILD-free at approximately day 99, but a steeper decline was observed after day 200, suggesting an increased risk of ILD between 90 and 200 days post-treatment (Figure 1).

### 3.2. Potential Risk Factors for ILD Development

Five potential risk factors were examined in association with ILD onset (Table 3). None of the examined factors (age, ground-glass opacities, hormone receptor status, Ki-67, HER2 IHC) reached statistical significance (all *p* > 0.05). These findings are exploratory only. The average time to ILD onset following the first dose of T-DXd was 189.2 ± 190 days, with a median onset of 125.5 days. The most frequently reported symptoms at ILD onset were dyspnea and cough (50%). Other symptoms included sore throat and upper respiratory discomfort. ILD severity was categorized using CTCAE criteria, with grades 1–2 considered mild and grades 3–5 considered severe. Symptom distribution did not differ significantly between severity groups (*p* = 0.377) (Table 4). Pulmonary function tests (PFTs) were performed in only 20% of ILD cases. In patients who developed ILD, the mean oxygen saturation at presentation was 95.20 ± 8.17%, suggesting a clinically relevant degree of hypoxia. Patients with Grade ≥ 3 ILD had lower SpO_2_ at presentation (median 88%, IQR [82–93]) compared with Grade ≤ 2 (median 98%, IQR [96–99]; *p* = 0.049). Median values are reported to avoid misinterpretation beyond the physiologic 100% limit (Table 4).

### 3.3. Management of ILD

**Figure 2:**
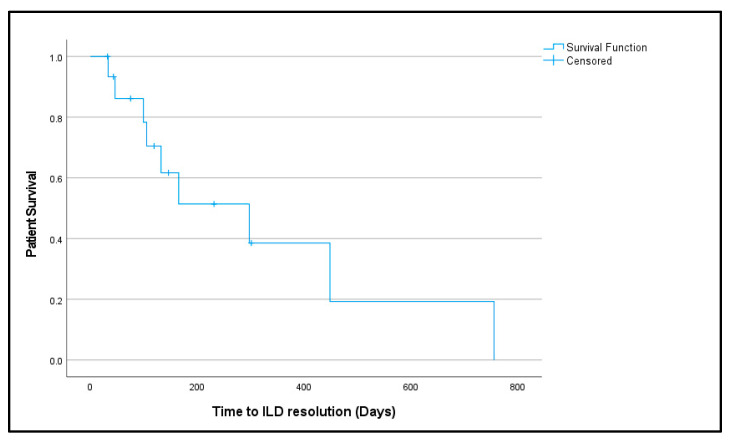
Survival (patients who attained resolution) over time.

Early detection, usually via symptom screening and pulse oximetry, was associated with higher SpO_2_ at diagnosis and a greater proportion of grade 1–2 ILD cases (*p* = 0.034), suggesting improved outcomes when ILD is recognized promptly. However, the timing of ILD identification (early vs. late) did not differ significantly between severe and mild ILD cases (Table 5). Therapeutic interventions—including corticosteroids alone, oxygen supplementation, and combination approaches—did not show statistically significant differences in outcomes (*p* > 0.05). Likewise, dose modification of T-DXd did not appear to impact ILD outcomes significantly (Table 5). Based on Kaplan–Meier analysis, the median time to ILD resolution was estimated at 297 days (95% CI: 77.46–516 days) (Figure 2). The resolution curve declined sharply within the first 200 days, indicating that most cases achieved clinical improvement within that period.

## 4. Discussion

In this real-world cohort, we found that ILD occurred in approximately one-quarter of patients receiving T-DXd, a rate noticeably higher than the 10–15% incidence reported in clinical trials and pooled analyses [10]. This disparity may be attributed to differences in patient populations and monitoring intensity between routine practice and clinical studies. Notably, earlier trials of T-DXd reported ILD in ~11% of patients (with ~2% experiencing fatal ILD) whereas subsequent trials with rigorous surveillance (such as scheduled imaging every 6 weeks) showed lower ILD rates and no grade 4–5 events [10,12]. It is also important to distinguish HER2-expressing tumors (HER2-positive or HER2-low) from HER2-mutant tumors, which are defined by ERBB2 genomic alterations. ILD incidence has been consistently higher in HER2-mutant NSCLC than in HER2-expressing breast cancers. As our cohort included only HER2-expressing cancers, direct comparisons with HER2-mutant populations should be interpreted cautiously. Such stringent monitoring is challenging to implement in everyday practice potentially leading to later recognition of ILD and a higher apparent incidence in real-world settings [12]. It is worth noting that certain populations appear more susceptible to T-DXd pneumonitis [10]. For example, HER2-mutant lung cancer patients have shown ILD rates around 24–26% likely due in part to underlying pulmonary comorbidities and diminished lung reserve [7]. Our finding of a 24.6% ILD incidence aligns with the upper range of these reports, underscoring the importance of contextual factors such as cancer type and baseline lung health in ILD risk assessment. Despite the relatively high incidence, our analysis did not identify definitive baseline predictors for ILD development. Traditional risk factors like smoking history, prior thoracic radiation, or presence of lung metastases were not significantly associated with ILD in our cohort. We observed a non-significant trend toward higher ILD odds in patients with pre-existing ground-glass opacities on imaging (OR~2.7), suggesting that underlying subclinical lung inflammation or fibrosis might predispose patients to drug-induced injury. This is biologically plausible and consistent with observations that patients with compromised lung parenchyma (whether from metastatic tumor, prior infections, or other interstitial changes) have a lower threshold for developing ILD [10]. Literature to date has highlighted a few factors that may elevate ILD risk with T-DXd, including East Asian ethnicity (particularly Japanese patients), reduced baseline oxygen saturation, and possibly higher drug exposure [12]. Interestingly, a recent pooled analysis indicated that younger patients (<65 years) had a higher likelihood of ILD than older patients [4]. This counterintuitive finding might reflect cohort effects for instance, older or frailer patients in trials could have been monitored more cautiously or discontinued therapy earlier, mitigating ILD detection. In our predominantly middle-aged (mean~49 years), female population (reflecting the HER2-positive breast cancer demographic), we were underpowered to draw firm conclusions on age or sex as risk modulators. Overall, the lack of clear-cut risk factors in our real-world data reinforces that all patients on T-DXd warrant vigilant pulmonary monitoring, not just those with obvious predispositions. The clinical presentation and time course of T-DXd-associated ILD in our study were in line with previously reported patterns. The median time to ILD onset was ~125 days (approximately four months) after T-DXd initiation, with most cases occurring within the first 6–7 months of therapy. Prior trials have reported a median onset ranging from about 3 to 6 months and a recent systematic review noted a median of ~193 days in one study [10]. This suggests that the risk is heaviest during the early to mid-period of treatment, though late-onset cases can occur. The majority of our patients presented with the hallmark symptoms of drug-induced ILD progressive dyspnea and cough which mirrors the clinical features described in trials and case series [10]. Importantly, we found that patients who developed severe ILD (CTCAE grade ≥ 3) had significantly lower oxygen saturation at presentation (mean~88%) compared to those with grade 1–2 ILD. While intuitive, this observation underscores a critical point: oxygen desaturation is a red flag for pneumonitis severity and should heighten clinical urgency. Indeed, one pooled analysis identified baseline SpO_2_ < 95% as a risk factor for ILD development, and our data suggest that even during ILD onset, SpO_2_ is a useful gauge of severity. Together, these findings support routine pulse oximetry assessments and prompt evaluation of any drop in oxygenation or new respiratory symptoms in patients on T-DXd [4]. Early symptoms can be nonspecific (e.g., mild cough or subtle exertional dyspnea), so maintaining a high index of suspicion is essential, especially since an initial mild ILD can rapidly worsen if T-DXd is continued unwittingly [13]. Our real-world management of ILD largely adhered to emerging best practices and recommendations from the literature. All patients with suspected ILD had T-DXd therapy interrupted at onset of pneumonitis, and the majority were started on systemic corticosteroids, consistent with standard management guidelines [11]. High-dose steroids (e.g., intravenous methylprednisolone) followed by a gradual taper are the cornerstone of ILD treatment, alongside supportive care such as supplemental oxygen for hypoxemic patients [11]. In our cohort, outcomes were generally favorable: most patients achieved clinical improvement or resolution of ILD with appropriate therapy, and we did not record any ILD-related deaths. This is a noteworthy finding considering that across clinical trials ~10% of ILD cases have led to fatal outcomes [10]. The absence of mortality in our series likely reflects heightened awareness and early intervention a “real-world” improvement that coincides with greater experience using T-DXd. It is also possible that milder cases were more readily recognized (owing to routine follow-ups and perhaps patient education to report symptoms early) and managed before progressing to acute respiratory distress. We observed a median time to ILD resolution of 297 days in our study, which is considerably longer than the roughly 34 days median recovery reported in clinical trials [10]. This discrepancy may be due to differences in the definition of “resolution” and the threshold for declaring a patient ILD-free. Trials often defined resolution as improvement sufficient to resume therapy or return to baseline respiratory status, whereas in practice we may have waited until near-complete radiographic and symptomatic resolution, often under a prolonged steroid taper [10]. It also reflects that some patients had chronic radiographic changes that were slow to fully normalize. Regardless, the prolonged course in some patients highlights the need for patience and cautious follow-up; premature re-challenge with T-DXd (if at all considered) should only occur once ILD is fully resolved and the patient is off steroids, given the risk of recurrence. Recent real-world analyses suggest that re-challenging patients after a mild ILD (grade 1) can be feasible and safe in select cases, but our institutional approach has been to permanently discontinue T-DXd for grade ≥ 2 ILD in accordance with current guidelines [14]. No patient in our series was re-exposed to T-DXd after an ILD event, so we cannot comment on the safety of re-challenge in our population. Collectively, our findings emphasize the value of proactive ILD surveillance and multidisciplinary management for patients on T-DXd. Oncologists, pulmonologists, radiologists, and pharmacists all play a role in mitigating this toxicity. Key practical measures include a thorough baseline assessment (history of lung disease, baseline imaging to identify any lung abnormalities), patient education to report symptoms early, and scheduled monitoring during therapy. In our center, we now obtain baseline high-resolution CT scans on all patients before starting T-DXd and consider repeating imaging if any respiratory symptoms develop, rather than waiting for symptoms to become severe. We also perform pulse oximetry at each clinic visit—an easily accessible tool that can uncover subclinical oxygen desaturation. While routine interval CT screening for asymptomatic ILD (as was performed every 6 weeks in clinical trials) is not always practical in standard practice due to cost and radiation concerns, a reasonable compromise is to maintain a low threshold for imaging [12]. In patients with even mild new cough or shortness of breath, especially in the first 6 months of therapy, prompt evaluation with CT chest can catch ILD at an early grade. By implementing such measures, real-world clinicians can approach the level of ILD vigilance seen in trials, ideally preventing the most devastating outcomes. Notably, our study adds to the growing body of evidence that early detection and management of ILD significantly improve patient safety on T-DXd. The primary limitations of our study are its retrospective design and relatively small sample size. With only 16 ILD events, the study may have been underpowered to detect modest risk factor associations; some findings (e.g., the impact of baseline ground-glass opacities) suggested a trend but did not reach statistical significance. Additionally, the identification of ILD cases depended on clinical documentation and the treating physicians’ judgment. It is possible that very mild subclinical ILD cases went unrecognized, or conversely that some reported ILD diagnoses might have reflected other etiologies (such as infectious pneumonitis) in the absence of biopsy confirmation. We tried to mitigate misclassification by requiring radiographic evidence or specialist assessment for ILD diagnosis, but about 15% of ILD cases in our series lacked dedicated high-resolution CT imaging, which could affect diagnostic certainty. Another limitation is the homogeneity of our patient population since the vast majority had HER2-positive breast cancer and were female, which limits the generalizability of our findings to other cancer types (e.g., gastric cancer or NSCLC) and to male patients. Nonetheless, given that breast cancer is the most common context for T-DXd use, our data remain highly relevant to current practice. Finally, as a single-center study from a tertiary hospital in the Middle East, our results should be extrapolated with caution to other settings; genetic and environmental factors (such as regional differences in ILD predisposition or pulmonary infectious exposures) might influence outcomes. Despite these limitations, our real-world analysis provides valuable insights by complementing clinical trial data with evidence from a less-selected patient cohort. It reinforces that ILD is not just a theoretical concern observed in trials, but a tangible risk in routine care that requires ongoing vigilance. Because T-DXd is dosed until progression or unacceptable toxicity, cumulative exposure may influence ILD risk. In our cohort, most ILD cases appeared between cycles 3–8, supporting heightened monitoring during this treatment window.

## 5. Conclusions

In this real-world cohort, ILD occurred in nearly one-quarter of patients receiving trastuzumab deruxtecan, mostly within the first six months. While outcomes were favorable with early intervention, the findings highlight the need for close monitoring—especially in patients with baseline lung abnormalities. Routine symptom checks, pulse oximetry, and timely imaging can support early detection. Continued research is needed to refine risk prediction and management strategies.

## Figures and Tables

**Table 1:** (**A**) Baseline patient demographics and cancer receptor status (*n* = 65); (**B**) Medical history, risk aspects, baseline organ functioning, and imaging characteristics.

**(A)**	
**Characteristic**	**Value**
**Demographics**	
**Age (years), mean ± SD (range)**	49 ± 11.82
**Gender, *n* (%)**	Male: 3 (4.6%) Female: 62 (95.4%)
**Weight (kg), mean ± SD**	63.37 ± 15.16
**Height (cm), mean ± SD**	156.76 ± 15.16
**BMI (kg/m^2^), mean ± SD**	25.78 ± 5.75
**Cancer and Receptor Status**	
**Cancer Diagnosis, *n* (%)**	Breast, 59 (90.8%) Ovarian, 1 (1.5%) Gastric, 2 (3.1%) Colon, 1 (1.5%) Endometrial, 2 (3.1%)
**Stage Group, *n* (%)**	Stage IV: 65 (100%)
**HER2-IHC Score, *n* (%)**	Positive, 36 (56.3%) Negative, 28 (43.8%)
**HER2-FISH Result, *n* (%)**	Positive, 16 (24.6%) Negative, 3 (4.6%) Unknown, 46 (70.8%)
**HER2 Status, *n* (%)**	Positive, 38 (58.5%) Low, 27 (41.5%)
**Progesterone Receptor Status, *n* (%)**	Positive, 31 (52.5%) Negative, 28 (47.5%)
**Estrogen Receptor Status, *n* (%)**	Positive, 40 (67.8%) Negative, 19 (32.2%)
**Ki-67 (%), mean ± SD**	41.68 ± 24.40
HER2-negative was defined as IHC 0. HER2-low included IHC 1+ or IHC 2+ with negative ISH. Ki-67 testing was performed as part of routine pathology and was available for most baseline biopsies; metastatic-site Ki-67 was not consistently assessed.	
**(B)**	
**Medical History and Risk Factors**	
**Smoking History, *n* (%)**	Yes, 1 (1.5%)
**History of Lung Radiation, *n* (%)**	Yes, 27 (41.5%)
**History of COPD/Emphysema, *n* (%)**	Yes, 4 (6.2%)
**History of Ground Glass Opacities, *n* (%)**	Yes, 39 (60.0%)
**Allergies or Contraindications, *n* (%)**	No known allergies, 44 (67.7%) Present allergies, 21 (32.3%)
**General Medical History, *n* (%) (prevalent ones)**	Hypertension, 12 (18.5%) Diabetes + hypertension, 3 (4.6%) Hypertension + hypothyroidism + dyslipidemia, 4 (6.1%)
**Baseline Organ Function and Imaging**	
**Sites of Metastasis, *n* (%)**	Lung/liver/brain, 25 (38.5%) Liver/Bone/Brain/Pancreas, 18 (27.7%) Bone, 9 (13.8%) Liver, 2 (3.1%) Peritoneum/spleen, 3 (4.6%) Lymph node/liver/lung, 6 (9.2%)
**Presence of Lung Metastasis, *n* (%)**	Yes, 33 (50.8%) No, 32 (49.2%)
**Respiratory Function Test Status, *n* (%)**	Abnormal, 2 (3.1%) Normal, 62 (95.4%)
**Requirement for Oxygenation, *n* (%)**	Yes, 10 (15.4%)
**Baseline SpO_2_ (%), mean ± SD**	98.09 ± 1.77
**Serum Creatinine (** μ **mol/L), mean ± SD**	55.26 ± 41.32

**Table 2:** Incidence of ILD with potential aspects influencing the rate of occurrence.

Aspects Influencing ILD Incidence		Developed ILD (*n*, %)	Did Not Develop ILD (*n*, %)	Total (*n*)	*p*-Value
**Lung radiation history**	Yes	8 (29.6%)	19 (70.4%)	27	0.429
	No	8 (21.1%)	30 (78.9%)	38	
**Lung metastasis presence**	Yes	9 (24.6%)	23 (71.9%)	32	0.518
	No	7 (21.2%)	26 (78.8%)	33	
**Smoking history**	Smoker	0 (0%)	1 (100%)	1	0.565
	Non-smoker	16 (25.0%)	48 (75%)	64	

**Table 3:** Logistic regression determining potential risk factors for ILD T-DXd-treated patients.

Variable	OR (95% CI)	*p*-Value
**Age (per year increase)**	1.03 (0.971–1.10)	0.306
**History of Ground Glass Opacities**	2.70 (0.522–14.01)	0.236
**Ki-67 (%)**	0.98 (0.95–1.02)	0.116
**HER2 IHC 3+ (vs. lower)**	0.71 (0.129–3.88)	0.690
**Estrogen Receptor status**	0.98 (0.95–1.02)	0.132
**Progesterone Receptor status**	4.02 (0.49–33.04)	0.196

**Table 4:** Difference in clinical presentation of ILD induced by T-DXd.

Variable		*p* Value
**Onset of ILD after T-DXd initiation, mean plus ±** **SD (median) days**	189.2 ± 190 (124.5)	0.187
**Symptoms associated with the onset of ILD, *n* (%)**	Shortness of breath + coughing, 8 (50.0%)	0.377
	Shortness of breath, 2 (12.5%)	
	Coughing + fever + sore throat, 1 (6.3%)	
	Shortness of breath + tachycardia + tachypnea, 1 (6.3%)	
	Asymptomatic, 2 (12.5%)	
	None, 2 (12.5%)	
**Pulmonary function test**	Performed: 20.0% Not performed: 80.0%	
**SpO_2_, mean ±** **SD (%)**	Mild ILD: 97.73 ± 2.41	0.049

**Table 5:** Managing T-DXd-induced ILD using early detection, dose modification, and therapeutic approaches.

Management	ILD Severity (n, %)			SpO_2_ (mean, SD)	
**Early detection**	Severe ILD	2 (50%)	0.182	97.82 ± 2.23	0.034
	Mild ILD	2 (16.7%)			
**Late detection**	Severe ILD	2 (50%)		88 ± 14.17	
	Mild ILD	10 (83.3%)			
**Dose modification (Hold days) mean, SD**	Severe	30	0.814		
	Mild	40.8 ± 41.25			
**Specific therapeutic interventions**	Prednisolone:	Severe = 16.7% Mild = 83.3%	0.178		
	Prednisolone + oxygen supplement	Severe = 60% Mild = 40%			
	Nasal cannula	Severe = 0% Mild = 100%			

Early detection = identification at first abnormal symptom/vital before significant hypoxemia.

## Data Availability

The datasets generated and analyzed during the current study are not publicly available due to patient confidentiality restrictions but are available from the corresponding author on reasonable request and with appropriate institutional approvals.
